# Advances in Physiological and Molecular Mechanisms of Heat Stress in Apple and Pear

**DOI:** 10.3390/plants15162429

**Published:** 2026-08-09

**Authors:** Gang Niu, Yue Yao, Longfei Li, Minghui Ji, Huan Liu, Lijuan Gao, Xumin Wang, Haijiao Xu, Da Zhang, Yingjie Wang, Jintao Xu, Baofeng Hao

**Affiliations:** Changli Institute of Pomology, Hebei Academy of Agriculture and Forestry Sciences, Qinhuangdao 066600, China; niug@nwafu.edu.cn (G.N.); shv266@163.com (L.L.); d.zhang@nwafu.edu.cn (D.Z.);

**Keywords:** apple, pear, gene expression regulation, thermotolerance mechanisms

## Abstract

Persistent global warming significantly impacts crop phenology and productivity, with perennial fruit trees facing heightened challenges due to their long life cycles and complex heat stress accumulation. Apple and pear, which hold substantial economic and nutritional value, are particularly vulnerable to high temperatures, manifesting as accelerated phenology, impaired floral organ development, disrupted pollination and fertilization, and insufficient fruit coloration—all of which severely compromise fruit quality and commercial value. Although recent advances have been made in elucidating heat stress signal transduction and regulatory networks in model plants such as Arabidopsis and rice, research on heat stress responses in apple and pear remains limited. This review systematically synthesizes the physiological responses, gene expression regulation, and protective cultivation strategies under high-temperature stress in apple and pear, aiming to provide a theoretical foundation for thermotolerance breeding and the establishment of heat stress regulatory networks, thereby supporting sustainable production in the context of global warming.

## 1. Introduction

Over the past several decades, the global mean surface temperature has risen at a relatively steady rate of approximately 0.19 °C per decade. This sustained warming has intensified the instability of the climate system, manifesting in a higher frequency and severity of extreme weather events—including heatwaves, droughts, and altered precipitation regimes—that increasingly disrupt agricultural systems. As a sector inherently sensitive to climatic conditions, agriculture has borne a disproportionate share of these impacts. In particular, rising temperatures have significantly shortened crop phenological phases and reduced vegetative growth rates, thereby constraining potential yields. These temperature-driven changes have emerged as a primary factor undermining global food supply stability [1]. Compared with herbaceous crops such as rice, fruit trees are perennial and long-lived, subjecting them to heat stress over extended periods and under more intricate conditions [2]. Persistent warming not only disrupts the phenological phases of dormancy and flowering in fruit trees, but also accelerates fruit ripening, which in turn leads to insufficient sugar accumulation, reduced individual fruit size, and overall quality deterioration. These changes collectively impair the commercial value of fruit produce [2].

Due to their widespread consumption, as well as their nutritional and economic value, apple and pear have become widely cultivated fruits worldwide and represent important commodities within the fruit crop industry. High-quality apples and pears serve as an important source of nutrients, vitamins, and bioactive compounds in the human diet [3]. Global warming poses a threat to the production of apple and pear. Model projections indicate that global surface temperatures will continue to rise throughout the 21st century, with marked regional disparities in the warming magnitude [4]. These climatic shifts exert complex, non-linear effects on major apple and pear production regions. Increasing heat resources are driving a significant poleward and upward expansion of suitable cultivation areas, whereas some traditional growing zones face the loss or relocation of viable conditions [5]. Concurrently, more frequent extreme weather events and phenological mismatches pose serious threats to yield and fruit quality. Insufficient winter chilling hours and heat stress during the growing season not only shorten dormancy but also reduce sugar content, diminish fruit size, and elevate the risk of sunburn [6]. Although warmer and wetter conditions may favour expansion in certain regions, they also markedly increase the compound risks of flowering frost and spring freeze events, thereby posing substantial challenges to maintaining stable apple and pear production [7]. As global warming continues to intensify, elucidating the molecular mechanisms underlying thermotolerance in apple and pear, and identifying key heat-tolerance genes are of strategic importance for developing new varieties adapted to future climatic conditions.

To cope with diverse types of high-temperature stress, plants have evolved sophisticated molecular regulatory networks that coordinate transcriptional reprogramming and physiological adjustments, ultimately driving adaptive morphogenesis [8]. Over the past few decades, the plant response to heat stress has garnered considerable research attention. Major advances in this field encompass the perception and transduction of heat signals, the interplay between heat shock responses and multi-hormonal signaling, the establishment of acquired thermotolerance, and the discovery of key thermotolerance-associated genes [2].

Compared with annual crops such as tomato, research on heat stress in apple and pear remains relatively scarce, particularly with respect to the elucidation of response mechanisms and the discovery of thermotolerance-related genes. Investigating thermotolerance in fruit trees not only provides a theoretical foundation for stress-resistant breeding and the construction of regulatory networks governing heat stress responses, but also holds practical significance for alleviating heat-induced damage and enhancing fruit yield. In this review, we systematically summarize the physiological responses and gene expression regulatory mechanisms of apple and pear under high-temperature stress, and further discuss protective strategies, the application of emerging technologies, and promising directions for future research.

## 2. Physiological Responses of Apple and Pear Under Heat Stress

### 2.1. Effects of Heat Stress on Photosynthesis

Temperature regimes profoundly affect key physiological processes in fruit trees, including transpiration, photosynthesis, and respiration, which can lead to insufficient supply of energy and carbohydrates, and consequently cause floral organ malformation or abortion [2]. The photosynthetic response of plants to heat stress is dependent on both the intensity and duration of the stress [9]. Under mild heat stress, enhanced photorespiration and reduced stomatal conductance result in a transient decline in photosynthetic rate. Under serious heat stress or heat shock, however, the loss of thylakoid membrane integrity and inactivation of photosystems lead to a primary decrease in leaf photosynthetic electron transport rate [10]. High temperature inhibits photosynthesis in both apple and pear, but apple exhibits relatively weaker heat tolerance and is more prone to leaf sunburn and fruit sunburn, whereas pear is relatively more heat-tolerant, although it also suffers from physiological stress under extreme high-temperature conditions [11]. Studies have shown that under high-temperature stress, the stomatal aperture of apple leaves is significantly reduced, while in pear leaves, the contents of chlorophyll, indole-3-acetic acid (IAA), and abscisic acid (ABA) decrease markedly. In contrast, malondialdehyde (MDA) content exhibits a continuous increase, and the maximum photochemical efficiency of photosystem II is significantly impaired [11,12,13,14]. However, most of these measurements were obtained from detached leaves or potted seedlings under controlled settings, leaving a critical gap in our understanding of photosynthetic responses in field-grown trees and, more importantly, in the comparative dynamics of heat-induced inhibition and recovery between apple and pear.

### 2.2. Effects of Heat Stress on Reproductive Systems

The reproductive phase represents one of the most heat-sensitive stages in fruit tree production, and high temperature affects the reproductive system through multiple pathways. Elevated temperatures critically impair flowering in both apple and pear, albeit with species-specific manifestations. In apple, heat stress primarily disrupts floral induction and curtails the flowering period [15]. In pear, however, floral buds are highly susceptible to abortion during differentiation, and its flowering phenology is subject to a more pronounced temperature-responsive regulatory network, involving the modulation of endogenous melatonin and the expression of dormancy-associated genes [16]. In pear, heat stress promotes early flowering and alters the expression of the light-harvesting complex protein gene *PpyLhcb4* as well as other flowering-related genes. Heterologous expression of PpyLhcb4 in Arabidopsis increased chlorophyll content and delayed flowering under both normal and high temperatures, suggesting that this gene suppresses flowering by enhancing chlorophyll biosynthesis [17]. Flowering induction in apple is also strongly influenced by ambient temperature: while 18–21 °C promotes induction, prolonged exposure to temperatures above 27 °C for several weeks exerts negative effects [15]. In grapevine and possibly other woody perennials, DELLA proteins act as positive regulators of flowering. The temperature-induced degradation of DELLA proteins appears to be conserved between apple and Arabidopsis, and degradation of MdRGL1a may underlie the heat-induced suppression of flowering in apple [18].

Pollen is particularly vulnerable to high temperature, which inhibits meiotic division of pollen mother cells, leading to pollen abortion, shrinkage, reduced viability and germination, and impaired anther dehiscence [19]. Following 72 h of pollination at 35 °C, apple pollen tubes exhibited morphological abnormalities such as swelling, bending, and nodule-like protrusions; style browning and callose deposition also occurred, preventing pollen tubes from entering ovules for fertilization [20]. These reproductive impairments ultimately result in substantial flower and fruit drop, and a marked decrease in fruit set rate [21]. Although these findings reveal species-specific disruptions in flowering and fertilization under heat stress, direct comparative data between apple and pear remain scarce, particularly for pollen-stage responses.

### 2.3. Effects of Heat Stress on Fruit Quality

Heat stress disrupts nutrient accumulation during fruit development, leading to retarded growth, reduced fruit size, abnormal ripening, and severely compromised quality. Apple exhibits a higher overall sensitivity to high temperature than pear, with heat injury being particularly pronounced in the form of fruit sunburn, fruit malformation, and inadequate pigmentation. In pear, by contrast, heat stress primarily results in the suppression of anthocyanin biosynthesis, flesh mealiness, and fluid exudation. Controlled temperature experiments demonstrated that heat treatment impairs apple fruit coloration, decreases pigment accumulation and the expression of related genes, lowers soluble solids content (SSC) and titratable acidity (TA), reduces fruit size, and increases the incidence of physiological disorders such as cracking and spotting [22]. Temperature treatments applied at different developmental stages revealed that fruit at the expansion stage exhibited greater tolerance to heat stress, whereas tolerance declined at the color-turning and maturation stages, accompanied by peel browning. This browning is attributed to heat-induced damage to cell membranes, which facilitates the contact between polyphenol oxidase (PPO) and phenolic substrates, resulting in enzymatic browning [11].

High temperature accelerates fruit respiratory metabolism, promoting the breakdown and consumption of organic acids such as malic acid and citric acid, which results in a marked decrease in fruit acidity [23]. In sand pear, high temperature may alter the expression of genes involved in sucrose synthesis and degradation, leading to abnormal sucrose accumulation. Simultaneously, heat stress inhibits photosynthesis, reducing the synthesis of photosynthetic products and dry matter accumulation [24]. Furthermore, high temperature reduces fruit firmness by approximately 22% and diminishes flesh crispness in sand pear. It also suppresses the expression of sorbitol dehydrogenase (SDH) and sorbitol transporter (SOT) genes, causing excessive sorbitol accumulation in intercellular spaces and impaired translocation, thereby inducing severe watercore disorder [24]. These combined effects significantly compromise fruit storability and increase the risk of peel and flesh diseases during postharvest storage [23]. The aroma of apple and pear fruits is primarily composed of esters, aldehydes, and alcohols. High temperature represses the expression of genes in the lipoxygenase (LOX) pathway, reducing the synthesis of key aroma precursors such as aldehydes and esters, while also accelerating fruit ripening and diminishing fruit freshness [25]. Long-term records indicate that over the past 30 years, apple fruit acidity, firmness, and watercore development have declined, changes likely attributable to global-warming-induced earlier flowering and elevated temperatures during the maturation period [26].

Although these findings comprehensively document heat-induced fruit quality impairment in both species, direct comparative evidence—particularly regarding stage-specific thermosensitivity and the integration of source-to-sink carbon allocation—remains lacking. The molecular basis of divergent phenotypes has not been systematically compared, and long-term field data are heavily skewed toward apple. Future studies should combine field phenotyping with developmental stage-resolved comparative analyses to establish a species-level framework for heat-induced quality loss.

### 2.4. Effects of Heat Stress on Root Systems

Heat stress affects fruit tree root systems through multiple pathways, including structural damage to root morphology, impaired water and nutrient uptake and transport, and alterations in the rhizosphere microenvironment. Experiments in potted pear demonstrated that heat stress directly damages root cells, causing massive mortality of fine roots in the shallow soil layer and root tip necrosis, which markedly reduces overall root volume and fine root density. Concurrently, it disrupts root water uptake capacity, leading to irreversible aboveground wilting and leaf necrosis [27]. High temperature intensifies canopy transpiration and increases water demand. Roots, acting as sensors of soil moisture and temperature, transmit signals of supply–demand imbalance to the shoot, triggering stomatal closure and other defensive responses. When root water uptake fails to keep pace with transpiration, the tree suffers from water imbalance [28]. Furthermore, high temperature alters the allocation of carbohydrates and amino acids in roots and disturbs lipid metabolism; this metabolic reprogramming may activate root heat stress response pathways [28]. High temperature reduces root respiration rate and ATP availability, directly suppressing the energy-demanding nitrogen assimilation pathway and markedly decreasing nitrogen uptake efficiency in roots [28,29]. High temperature further alters rhizosphere microbial community structure. Although some plants can recruit beneficial microbes by secreting specific metabolites under heat stress to enhance resilience [28], hot and humid or drought conditions may also favor the proliferation of soil-borne pathogens such as those causing root rot, thereby exacerbating root disease incidence.

Although pear roots generally distribute more deeply than those of apple, root growth in both species is inhibited under persistent heat stress, and may even undergo necrosis. However, it remains uncertain whether the temperature thresholds that trigger growth inhibition and functional impairment of roots differ markedly between apple and pear. Comparative root-level heat response studies remain conspicuously underrepresented in the literature on both apple and pear, addressing this deficit is essential for informing rootstock selection and irrigation scheduling under future climate scenarios.

## 3. Mechanisms of Heat Stress Response

The physiological deterioration described above is underpinned by specific molecular events in each case. The following sections will elaborate on the mechanisms of heat signal transduction, transcriptional regulation of anthocyanin biosynthesis, the heat shock protein-mediated chaperone network, and epigenetic regulation under heat stress—processes that collectively orchestrate the heat-induced quality decline in apple and pear fruit. Notably, the molecular basis of fruit quality deterioration in these two species exhibits both conserved features and species-specific divergences, as summarized in Section 3.9 and Table 1.

### 3.1. Perception of High-Temperature Signals

Plants rely on intricate signaling networks to convert physical heat stimuli into biological instructions. The plasma membrane serves as an early site of heat perception, where elevated temperatures alter lipid composition and fluidity, inducing conformational changes or rapid phosphorylation of membrane receptors, thereby translating thermal signals into chemical cues [30]. Notably, the receptor kinase FERONIA functions as a thermosensory switch by dynamically assembling sterol-dependent plasma membrane nanoclusters, enabling the distinction between permissive growth temperatures and noxious heat, and subsequently triggering appropriate thermotolerance responses [30]. In rice, heat-induced disruption of plasma membrane lipid bilayers activates diacylglycerol kinase 7 (DGK7), which phosphorylates diacylglycerol (DAG) to generate phosphatidic acid (PA). PA then acts as a second messenger to initiate signal transduction, converting physical heat signals into lipid-based signals [31]. Membrane-localized receptor proteins, which are conserved thermosensors across prokaryotes and eukaryotes, perceive temperature changes likely through conformational rearrangements or activity modulation, often triggering Ca^2+^ influx or downstream post-translational modifications, ultimately enabling systemic integration of heat signals throughout the plant [32]. While direct evidence for heat perception mechanisms in apple and pear is lacking, the conservation of FERONIA-like receptors and DGK homologs in the apple genomes suggests similar thermosensory modules. However, functional validation remains a priority [33,34].

### 3.2. Heat Signal Transduction

Calcium ions (Ca^2+^), reactive oxygen species (ROS), and phytohormones serve as key signaling molecules in transmitting heat signals. Upon heat stress, the receptor-like kinase BAM1 is rapidly activated and recruits the cytosolic kinase PBS1 to form the BAM1-PBS1 complex, which subsequently phosphorylates RBOHD to trigger ROS production. ROS signaling further promotes Ca^2+^ influx, and the resulting Ca^2+^-calmodulin (CaM) complex activates downstream calcium-dependent protein kinases (CDPKs) or CaM-binding transcription factors, ultimately modulating the activity of heat shock transcription factors (HSFs) [32]. In Arabidopsis, the shoot apical meristem senses elevated temperature and generates a burst of nitric oxide (NO), which is converted into GSNO (S-nitrosoglutathione) as a mobile signal that is systemically transported through the vascular system [1]. In addition, elevated CO_2_ levels have been shown to partially alleviate heat-induced damage in plants. Recent work in tomato has identified βCA2 as a pivotal sensor that integrates CO_2_ and heat signals; its specific expression and plasma membrane localization under combined stress are essential for its signaling function. The receptor kinase PSKR1 phosphorylates βCA2 to coordinate CO_2_ and heat signaling and to modulate phosphatidic acid accumulation, thereby enhancing thermotolerance [35]. Transcriptomic evidence from pear roots indicates that calcium signaling genes are differentially expressed under heat, implying that Ca^2+^-mediated transduction pathways are operational in pear [36]. Whether similar dynamics occur in apple rootstocks has not been examined.

### 3.3. Role of ROS in Heat Stress Responses

ROS play a dual role in plant responses to high temperature: at low concentrations, they act as signaling molecules mediating early warning, signal transduction, and defensive adaptation; at high concentrations, they cause oxidative damage, disrupting cellular structure and function [37]. Heat stress perturbs cellular redox homeostasis, leading to compartment-specific accumulation of ROS in chloroplasts, mitochondria, and the plasma membrane. As second messengers, ROS activate MAPK cascades and calcium signaling pathways via oxidative modifications, transmitting stress signals to the nucleus and inducing the expression of heat shock proteins (HSPs) and antioxidant enzymes, thereby contributing to the establishment of thermotolerance [38]. However, excessive ROS accumulation triggers membrane lipid peroxidation, compromises membrane and organelle integrity, causes electrolyte leakage, and exacerbates cellular injury. It also induces endoplasmic reticulum stress, resulting in protein misfolding and programmed cell death (PCD), which ultimately leads to abortion of reproductive organs such as anthers and florets, as well as male sterility, severely reducing crop yield [37]. In kiwifruit, AcEGY interacts with AcCSD2 to promote H_2_O_2_ production in chloroplasts under heat stress. The generated H_2_O_2_ is released into the cytosol, where it induces oligomerization of AcHSFA2-2, enhancing its transcriptional activity and subsequently activating downstream heat-tolerance genes such as HSF70-2 and HSF20, ultimately improving kiwifruit thermotolerance [39]. In pear, H_2_O_2_ serves as a key signaling molecule that is widely involved in dormancy and germination, anthocyanin accumulation under high-light stress, and disease resistance [40,41]. In apple, ROS also participates in the responses to multiple stresses, including salt, drought, and disease [42,43,44].

### 3.4. Roles of Phytohormone Signaling in Heat Stress Response

In response to heat stress, phytohormones act as key signaling molecules that integrate environmental cues, regulate physiological responses, and establish thermotolerance. Heat stress induces auxin accumulation, which promotes Aux/IAA degradation and upregulates HSP transcription [45]. ABA maintains water balance by regulating stomatal closure and induces HSP expression [46]. Ethylene signaling activates heat-responsive genes through transcription factor cascades such as EIN3-ERF, thereby participating in the establishment of thermotolerance [47]. Jasmonic acid (JA), often in coordination with temperature sensors such as phyB, balances growth and stress adaptation through MYC2 and protects chloroplasts [48]. Brassinosteroid (BR) deficiency aggravates photoinhibition, whereas exogenous BR alleviates it [33,49]. Salicylic acid (SA) activates HSF to induce HSP transcription [50,51]. Gibberellin (GA) enhances thermotolerance by relieving DELLA-mediated repression of PIF4, which subsequently upregulates HSPs via NAC019 in Arabidopsis [52]. Transcriptome sequencing of *Pyrus betulifolia* under heat stress revealed significant enrichment of BR biosynthesis, arginine metabolism, and proline metabolism pathways; ABA, BR, methyl jasmonate (MeJA), and ethylene (ET) are all widely involved in the heat stress response of pear [53]. In apple, heat stress markedly increased the concentrations of ABA, JA, SA, and ET in the peel, and these hormones act synergistically to regulate fruit defense against heat and photooxidative stress [54].

Current evidence implicating hormonal regulation in heat responses of pear and apple is predominantly correlational, derived primarily from transcriptomic profiling and phytohormone measurements, and lacks functional genetic validation. Whether the well-characterized Arabidopsis signaling modules are conserved in these fruit tree species remains an open question. Moreover, the proposed synergistic interactions among hormones are not mechanistically substantiated. Future investigations should prioritize functional genetic approaches, incorporate multiple cultivars, and evaluate responses under environmentally realistic stress conditions to establish causal relationships.

### 3.5. Regulatory Mechanism of Anthocyanin Biosynthesis Under Heat Stress

**Figure 1:**
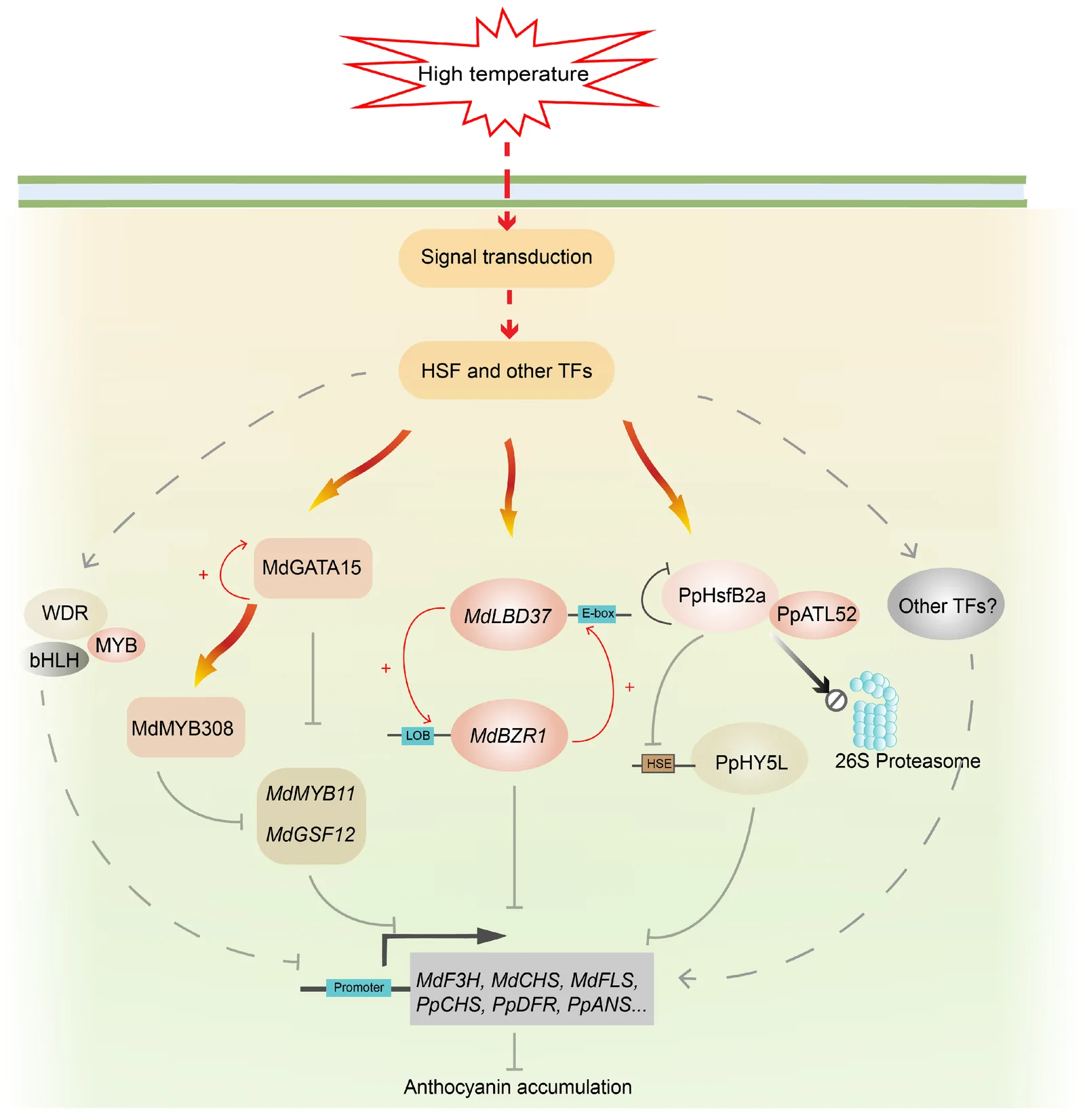
Regulatory mechanism of anthocyanin biosynthesis in apple and pear under heat stress. Red arrows and plus signs (+) denote transcriptional activation; The gray “stop sign” represents prohibition. Gray solid arrows indicate a decrease, while gray dashed arrows represent unverified alternative pathways.

Temperature exerts significant effects on anthocyanin biosynthesis in various fruit tree species [2]. High-temperature treatment suppressed the expression of three putative flavonoid biosynthetic genes—A5C (chalcone synthase, CHS), B5F (flavanone 3-hydroxylase, F3H), and D1F (flavonol synthase, FLS)—in apple (Figure 1) [55]. Both apples and pears rely on the highly conserved MYB-bHLH-WD40 (MBW) transcription factor complex as the core regulatory switch for anthocyanin biosynthesis [56]. In addition to the MBW complex, the B-class GATA transcription factor MdGATA15 has been identified as a heat-inducible regulator that exerts a complex modulatory effect on anthocyanin accumulation in apple peel [57]. MdGATA15 reduces anthocyanin accumulation by repressing the expression of multiple transcription factors, including *MdMYB11*, *MdANS*, and *MdGSTF12*. Concurrently, MdGATA15 enhances this suppressive effect by activating the expression of *MdMYB308*, a negative regulator of anthocyanin biosynthesis. Furthermore, MdGATA15 activates its own promoter through positive feedback regulation, thereby enhancing the stability of its expression [57]. The transcription factor *MdLBD37* has been shown to respond to high-temperature stress and suppress the expression of anthocyanin structural genes in apple [58]. A recent study further reveals that MdLBD37 binds to the lateral organ boundaries (LOB) element in the promoter of *MdBZR1*, forming a MdBZR1-MdLBD37 positive feedback regulatory loop that further inhibits anthocyanin biosynthesis in apple fruit [59]. The pear heat shock factor PpHsfB2a suppresses the transcription of *PpHY5L*, a key activator of light-regulated anthocyanin biosynthesis, by binding to the heat shock element in the *PpHY5L* promoter, thereby ultimately inhibiting anthocyanin accumulation. Under high temperature conditions, the abundance of PpHsfB2a protein is significantly increased due to reduced degradation via the proteasome pathway mediated by the RING-type E3 ubiquitin ligase PpATL52, which in turn diminishes anthocyanin production [60].

### 3.6. Heat Shock Proteins and Heat Shock Factors

High temperatures can induce protein misfolding or impair the protein folding process, thereby leading to cellular apoptosis. HSPs, acting as molecular chaperones, are involved in the refolding or degradation of misfolded proteins, and are also closely associated with plant stress tolerance [2,61]. The apple chloroplast-localized elongation factor MdEF-Tu positively regulates plant tolerance to heat stress. Under high-temperature conditions, MdEF-Tu exists in the form of insoluble aggregates and undergoes ubiquitination modification. At the protein level, MdEF-Tu interacts with a chaperone heat shock protein MdHsp70, which prevents the accumulation of ubiquitinated MdEF-Tu aggregates, thereby enhancing plant thermotolerance [61]. Heat treatment of apple tissue culture seedlings revealed that the HSF *MdHSFA2* was significantly induced over time. MdHSFA2 was further shown to activate transcription of the galactinol synthase gene *MdGoIS4*, and Arabidopsis lines overexpressing MdGoIS4 exhibited enhanced thermotolerance [62]. Quantitative PCR and transcriptomic analysis revealed that multiple HSP genes, including 12 *HSP20* family genes and 4 *HSP90* family genes, were upregulated in apple following high-temperature treatment. Among them, the *HSP20* family gene *MsHsp16.9* was shown to alleviate severe stress responses by positively modulating antioxidant enzyme activities and both ABA-dependent and ABA-independent signaling pathways [63,64,65,66]. The transcription factor MdWRKY75 in apple responds to multiple stresses [67]. Its expression is transcriptionally upregulated by heat stress, and overexpression of MdWRKY75 conferred enhanced thermotolerance in apple. Further experiments demonstrated that MdWRKY75 positively regulates the expression of the heat shock transcription factors *MdHSF4*, *MdHSFB2a*, and *MdHSFA1d*, thereby contributing to thermotolerance, whereas this regulation is suppressed by the heat shock protein MdHSC70 [67]. Transcriptomic analysis revealed that high temperature induced significant changes in gene expression in sand pear, with five HSF genes and 34 HSP genes being significantly upregulated [24]. Temperature experiments demonstrated that 39 °C represents a critical threshold for pear cells, effectively inducing the expression of heat shock proteins to mitigate cellular damage. In contrast, extreme temperatures of 42 °C and above exceed the cellular protective capacity, resulting in impaired protein synthesis and enhanced protein degradation, thereby causing severe cellular injury [68].

### 3.7. Other Genes Induced by High Temperature in Apple and Pear

In addition to HSF and HSP, heat stress also transcriptionally induces a diverse array of other genes. The apple nucleoporin gene *MdNup62* was significantly upregulated upon heat treatment. Transgenic Arabidopsis and tomato lines overexpressing *MdNup62* exhibited markedly reduced thermotolerance. Furthermore, MdNup62 interacts with multiple heat shock factors during nucleocytoplasmic transport, thereby regulating flowering and heat tolerance in apple [69]. Proteins containing the VQ motif are transcriptional regulators involved in plant growth and development. Transgenic apple plants overexpressing the VQ motif-encoding gene *MdVQ37* exhibited a heat-sensitive phenotype under high-temperature stress. Experimental evidence indicates that MdVQ37 functions in basal thermotolerance by modulating transcription factor activity and SA homeostasis [12]. Under high-temperature stress at 42 °C, the expression of the pear calcineurin B-like protein (CBL) genes *PbCBL1*, *PbCBL3*, and *PbCBL4* was downregulated, whereas *PbCBL2* expression was upregulated, reaching its peak at 6 h. These findings suggest that *PbCBL2* may play a crucial role in the response of pear to high-temperature stress [70]. Following treatment with salt stress (200 mM NaCl) and heat stress (40 °C), the expression levels of the pear ferritin genes *PpFer1*, *PpFer3*, and *PpFer4* were significantly upregulated, suggesting that these ferritin genes may play important roles in the adaptation of pear to salt and oxidative stress [71]. Proline-rich proteins (PRPs) are cell wall proteins involved in plant development and stress responses. The apple PRP-encoding gene *MdPRP6* is significantly upregulated under heat stress, and its overexpression in tobacco enhances high-temperature tolerance, suggesting that *MdPRP6* may serve as a key gene for thermotolerance in apple [72]. Additionally, multiple genes have been reported to be induced by heat stress, including the apple cell cycle inhibitor genes *MdWEE1*, *MdRBR*, and *MdKRPS*, the apple Argonaute (AGO) gene, suggesting their potential involvement in the response to high-temperature stress (Table 1) [73,74,75].

### 3.8. Epigenetic Regulation

**Figure 2:**
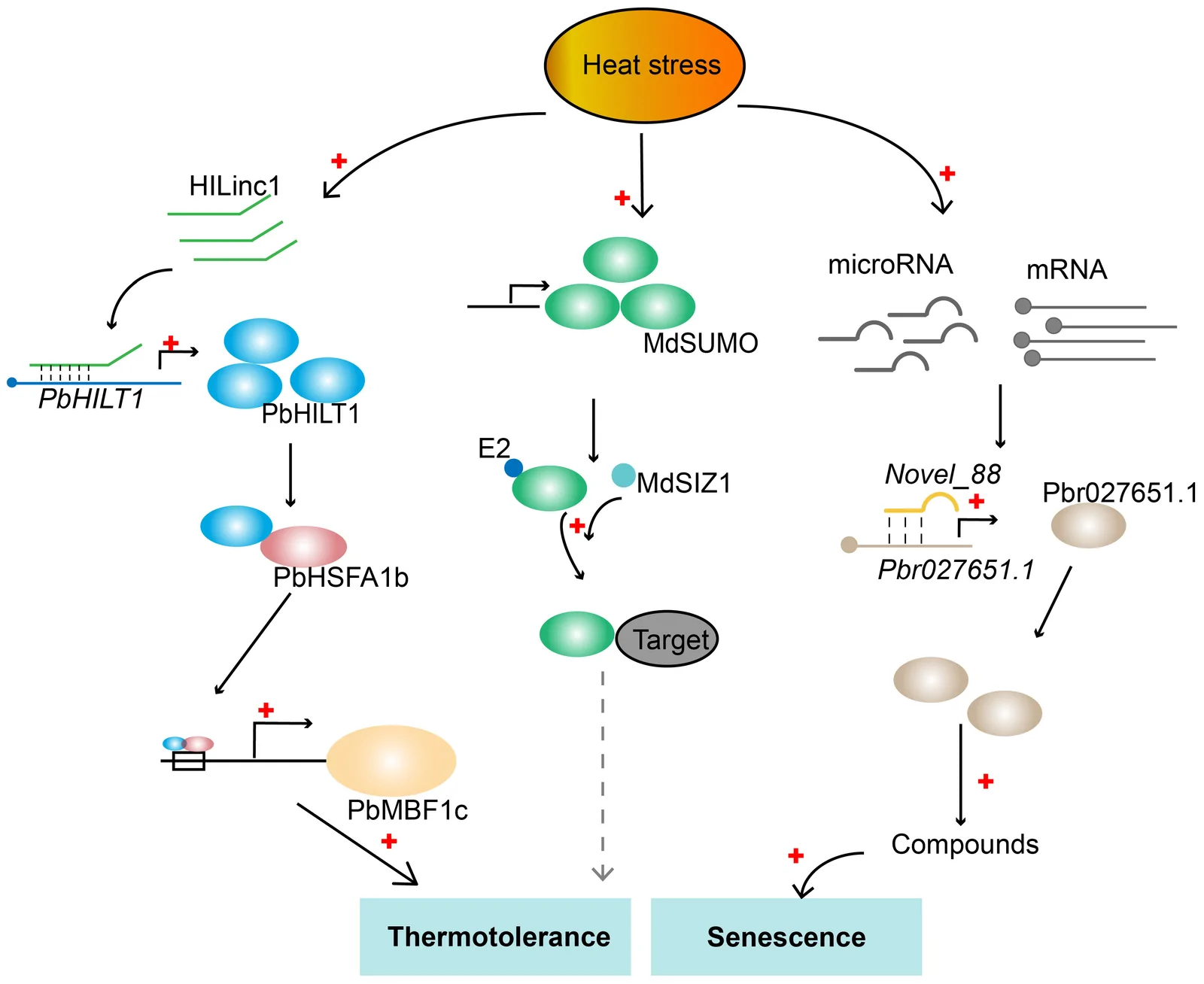
Schematic diagram of epigenetic regulation in apple and pear under heat stress. The symbol “+” indicates promotion, and all dashed lines represent that have not yet been experimentally confirmed.

High-temperature stress not only directly affects gene expression through transcriptional regulation but also regulates the expression levels of related genes by modulating epigenetic mechanisms (Figure 2). HILinc1 is a cytoplasm-enriched long non-coding RNA induced by heat stress and plays a critical role in thermotolerance regulation in pear. HILinc1 stabilizes the upregulation of its target gene *PbHILT1* through complementary base pairing. PbHILT1 interacts with heat shock transcription factor PbHSFA1b to enhance its transcriptional activity, thereby upregulating the downstream transcriptional regulator *PbMBF1c* during heat stress and consequently improving thermotolerance in pear [76]. SUMOylation, as a post-translational modification, participates in plant heat stress responses in a broad and rapid manner. There are six SUMO isoforms distributed across the six chromosomes of the apple genome, all of which are upregulated under high-temperature stress conditions. Transgenic apple plants with silenced MdSUMO2 exhibited higher survival rates under heat stress and accumulated more of the substrate protein MdDREB2A, which in turn induced the expression of heat-responsive genes, including *MdHSFA3*, *MdHSP26.5*, *MdHSP18.2*, *MdHSP70*, *MdCYP18-1*, and *MdTLP1*, thereby enhancing thermotolerance [77]. The apple SUMO E3 ligase MdSIZ1 exhibits SUMO E3 ligase activity both in vitro and in vivo. SUMO conjugation is enhanced by high temperature, low temperature, and ABA. Heterologous expression in Arabidopsis demonstrated that MdSIZ1-mediated SUMOylation of target proteins is a critical process enabling apple plants to adapt to diverse environmental stresses [78]. N6-methyladenosine (m6A) demethylases play a critical role in regulating plant responses to environmental stress. MdALKBH1A functions as an m^6^A demethylase in apple. Transgenic tomato plants overexpressing *MdALKBH1A* exhibited increased sensitivity to high temperature, likely due to reduced antioxidant capacity, enhanced membrane lipid peroxidation, and compromised plasma membrane stability [79]. Inappropriate high temperatures during fruit storage accelerate fruit senescence. Multi-omics analysis revealed that high temperature promoted the biosynthesis of type V and VI pro-senescence compounds while suppressing the accumulation of type II and III anti-senescence compounds by modulating the expression of 202 mRNAs in pear fruit. A total of 16 microRNAs responsive to high or low temperature were found to interact with senescence-related mRNAs. Among them, the microRNA Novel_188 was shown to mediate fruit senescence through interaction with its target Pbr027651.1 [80].

Although the studies summarized above have uncovered multiple molecular components and response mechanisms involved in heat tolerance in apple and pear, several limitations remain. Most functional evidence is derived from overexpression experiments in heterologous systems, while stable transgenic validation in apple or pear is still scarce. The crosstalk and hierarchical coordination among the various identified transcriptional regulatory modules remain poorly defined. Moreover, most experiments have been performed using tissue-cultured seedlings or detached materials under controlled conditions, leaving it unclear whether these mechanisms are applicable to field-grown fruit at commercial maturity. Finally, the majority of studies have focused on short-term heat exposure, whereas the effects of temperature fluctuations and recovery phases on anthocyanin stability, as well as the dynamic nature of epigenetic modifications, have received limited attention.

### 3.9. Comparative Analysis of Heat Stress Responses in Apple and Pear

Comparative analysis of heat responses in apple and pear reveals both conserved strategies and species-specific regulatory divergence. Both apple and pear contain the HY5-BBX core module and the COP1-HY5-mediated temperature-response pathway in their anthocyanin biosynthesis networks [56]. However, the downstream light-signaling pathways diverge considerably between the two species. In apple, MdBBX37 functions as a negative regulator by repressing MdHY5 expression and interfering with the binding of MdMYB1 to its target genes, thereby limiting anthocyanin accumulation. In contrast, in pear, PpBBX16/18 form a complex with PpHY5 that directly enhances the promoter activity of PpMYB10, acting as positive regulators and promoting anthocyanin production. These opposing regulatory modes result in fundamentally different outcomes for anthocyanin synthesis under light signals [56]. Regarding internal fruit quality deterioration, apple primarily exhibits enzymatic browning, associated with increased *MdPPO1* expression and elevated oxidative markers, whereas pear displays pronounced water core disorder [21]. In pear, heat upregulates sucrose synthase genes (SPS) while suppressing SDH and SOT, leading to sorbitol accumulation and impaired transport—a metabolic imbalance that distinguishes pear from apple [23]. Both species, however, share reliance on HSPs and HSFs for protein homeostasis. At the epigenetic level, both species exhibit sophisticated post-transcriptional and post-translational controls, albeit through different machineries. Apple utilizes SUMOylation via MdSIZ1 and the m^6^A demethylase MdALKBH1A to fine-tune heat responses, whereas pear employs a lncRNA module (HILinc1-PbHILT1-PbHSFA1b) and miRNA-mediated regulation of senescence-associated genes (Table 1) [76,77,78,79,80].

These divergences suggest that heat tolerance strategies are shaped by each species’ particular evolutionary history and ecological niche, and underscore the need for species-tailored breeding and management approaches. Despite these insights, direct comparative studies with standardized experimental conditions are scarce. Most findings originate from independent studies with disparate temperature regimes, developmental stages, and cultivars, making it difficult to ascertain whether observed differences truly reflect species-specific mechanisms or arise from methodological heterogeneity. Future research should prioritize parallel comparative experiments using the same stress protocols across multiple apple and pear cultivars.

## 4. Thermotolerance Mechanisms and Protective Measures Under Heat Stress

### 4.1. Antioxidant Systems in Heat Stress Responses

Under high-temperature stress, fruit trees suffer from excessive accumulation of ROS, leading to membrane lipid peroxidation, protein denaturation, and chloroplast damage, which severely impair growth, yield, and fruit quality. To counteract this, trees activate their intrinsic antioxidant defense systems, significantly elevating the activities of various antioxidant enzymes that synergistically scavenge superoxide anions and H_2_O_2_, thereby alleviating oxidative injury [81]. In grapevine, a WRKY transcription factor, TTC4, is upregulated upon heat stress and directly induces the expression of the heat shock protein gene *HSP18.1* and the antioxidant enzyme gene *APX3*, contributing to thermotolerance [82]. In apple, the HD-Zip protein MdHB7 functions as a positive regulator by directly binding to and activating the promoters of *MdPRX66* and *MdLac17*, which promotes ROS scavenging and lignin accumulation, thus enhancing basal heat tolerance [83]. Moreover, the transcript level of the autophagy-related gene *MdATG18a* is induced by high temperature; its overexpression elevates autophagic activity and reinforces the antioxidant system, reducing ROS-induced damage in apple plants [81].

### 4.2. Root Thermotolerance Regulatory Mechanisms

Heat stress reprograms root cellular processes, involving crosstalk among genes, phytohormones, ROS, and antioxidants. The spatiotemporal regulation and long-distance transport of auxin, cytokinin, and ABA are critical determinants of root growth and development under elevated temperatures [28]. Under heat stress, roots increase ABA biosynthesis or its translocation to the shoot. In cucumber, root-zone heat stress enhances ABA delivery to the canopy, which induces H_2_O_2_ accumulation in leaves and subsequently triggers the expression of HSP70, thereby improving canopy thermotolerance [84]. In longan, both heat stress and IAA induce the expression of the ERF transcription factor DlERF6, which enhances thermotolerance by promoting IAA biosynthesis and ROS scavenging [85].

Rhizosphere microorganisms assist plants in coping with abiotic stresses. Root exudates play a critical role in recruiting beneficial microbes that enhance stress resilience. In *Panax notoginseng*, heat stress induces the secretion of nucleotide compounds, which promote the proliferation of two beneficial strains, *Burkholderia* sp. and *Saitozyma podzolica*. Together, they synergistically increase superoxide dismutase (SOD) activity, thereby improving both thermotolerance and disease resistance [29]. Additionally, volatile organic compounds (VOCs) emitted by *Bacillus zanthoxyli* HS1 alleviate heat and salt stress in cucumber by reducing stomatal aperture, inducing callose deposition, and enhancing antioxidant enzyme activities [86].

### 4.3. Thermopriming

The phenomenon whereby a brief, non-lethal heat exposure confers enhanced tolerance to subsequent severe heat stress is termed thermopriming [87]. Primed plants reprogram energy metabolism, increasing branched-chain amino acids and lipid catabolites that serve as osmolytes and antioxidants, facilitating recovery and protecting membrane integrity [88]. Early during priming, BR signaling and glutathione metabolism are rapidly activated, promoting lignin deposition for structural reinforcement [89]. Epigenetic modifications, particularly histone demethylation, play a central role in establishing and maintaining heat stress memory. In Arabidopsis, Jumonji (JMJ)-mediated demethylation removes the repressive histone mark H3K27me3 at HSP loci, enabling faster transcriptional reactivation upon subsequent stress [89], additionally, de-repression of splicing in primed plants ensures proper processing of heat-responsive transcripts during a second heat event [90]. Studies on kiwifruit plantlets confirmed that moderate thermopriming effectively suppresses chlorophyll degradation and membrane permeability increase under high temperature, maintains low H_2_O_2_ levels, and significantly enhances thermotolerance [91].

Heat acclimation also mitigates the detrimental effects of heat stress on roots. In Arabidopsis, acclimation reduces the levels of NADPH oxidase and ROS, as well as the accumulation of ascorbate peroxidase and peroxidase, thereby alleviating root damage. Root acclimation involves crosstalk between antioxidant mechanisms and phytohormones—particularly ABA, ET, and cytokinin (CK)—to facilitate efficient growth recovery following heat shock release [28].

### 4.4. Protective Measures Under Heat Stress

**Figure 3:**
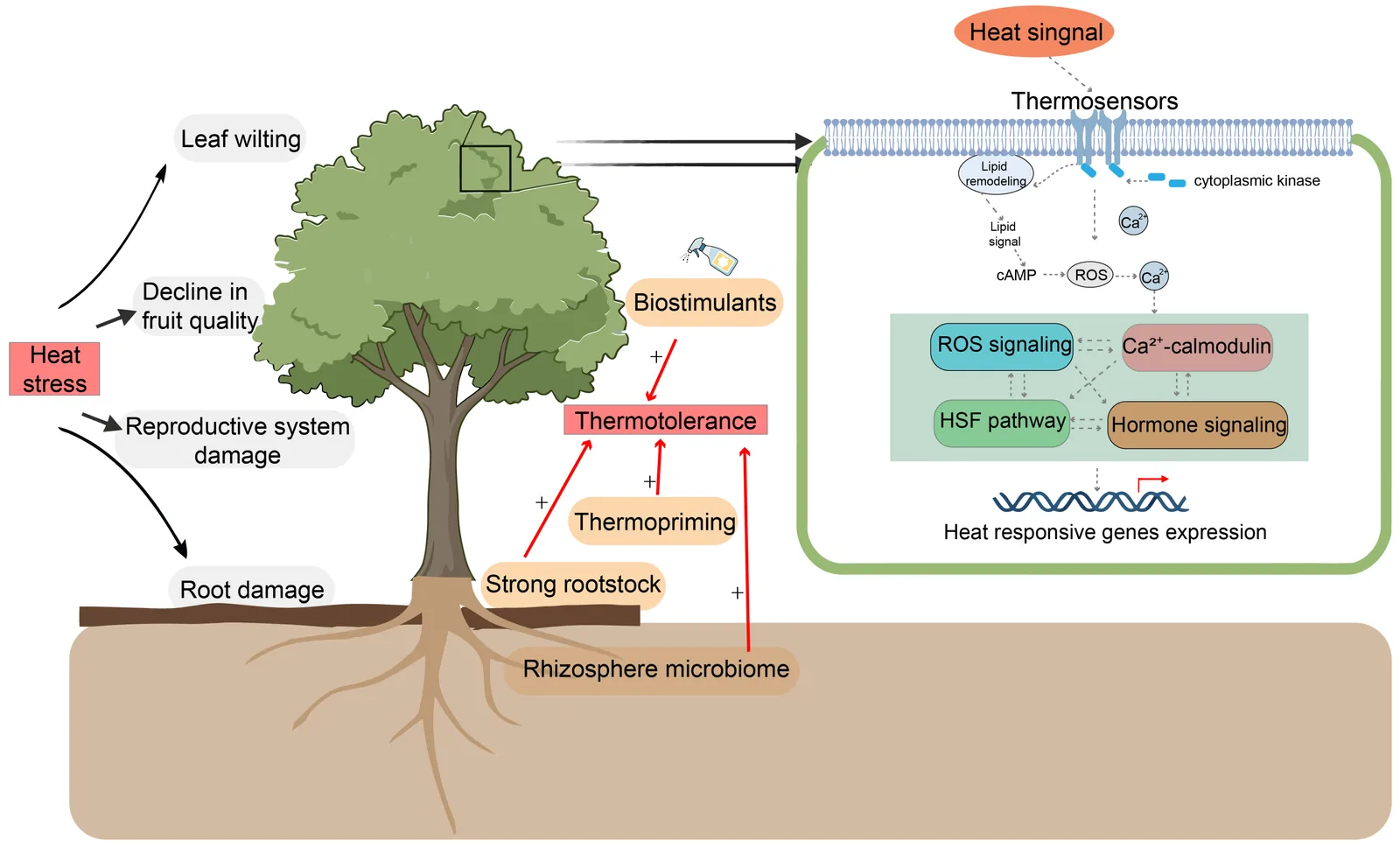
Schematic diagram of potential future directions in heat stress research on apples and pears. The symbol “+” indicates promotion, and all dashed lines represent that have not yet been experimentally confirmed.

In addition to heat acclimation, foliar application of phytohormones and plant biostimulants has proven effective in alleviating heat stress in fruit trees [92]. Heat stress disrupts chloroplast structure and suppresses photosynthesis. Biostimulants—such as seaweed extracts, amino acids, and protein hydrolysates—enhance the photochemical efficiency of photosystem II (PSII) and reduce thermal energy dissipation (Figure 3) [93]. They also modulate the ABA and CK levels, thereby promoting stomatal regulation and root protection under stress. Furthermore, in coordination with signaling molecules like SA, NO, and JA, biostimulants induce phenylpropanoid and flavonoid biosynthesis pathways, contributing to improved heat tolerance [93].

Studies have shown that exogenous application of SA and ascorbic acid (AsA) alleviates high temperature-induced oxidative stress at the photosynthetic level, thereby exerting positive effects on photosynthesis in apple leaves under high-temperature conditions [94]. *MdMIPS1* encodes a key rate-limiting enzyme in inositol biosynthesis and is highly expressed under high-temperature stress. Exogenous application of 100 μmol·L^−1^ inositol significantly alleviated high temperature-induced damage in apple plants [95]. Melatonin (MT) is a multifunctional signaling molecule. Both exogenous MT application and overexpression of the melatonin biosynthesis gene *MdASMT9* enhance autophagic activity by promoting MdWRKY33-mediated transcriptional upregulation of *MdATG18a* under heat stress, thereby improving thermotolerance in apple plants [96]. Under high-temperature and drought conditions, overhead irrigation for cooling, protective netting over the canopy, and protective spray formulations containing carnauba wax, modified clay, and calcium carbonate have been proven effective in preventing or reducing sunburn in apples [97]. Foliar application of plant phenols and quick-release phenols can also serve as part of an efficient and sustainable fertilization program for apple and pear trees to avoid the negative effects of abiotic stress [98]. Beyond protective measures such as spraying, the selection of rootstocks has been shown to alleviate the effects of heat stress. Rootstocks with larger root systems exhibit greater vigor and higher water transport capacity, resulting in a lower incidence of heat-damaged fruits and a higher marketable yield of healthy fruits [99].

## 5. Discussion and Prospect

### 5.1. Application of New Technologies in Mining Heat-Tolerance Genes

Global warming has established extreme heat as a major abiotic stress constraining crop yield and quality. Conventional forward genetics approaches are inherently limited in dissecting complex quantitative traits. However, the advent of multi-omics platforms—encompassing genomics, transcriptomics, proteomics, metabolomics, and single-cell sequencing—coupled with CRISPR/Cas9-based gene editing, has provided a transformative paradigm for pinpointing thermotolerance genes, unraveling molecular mechanisms, and engineering resilient germplasm [2].

GWAS and QTL mapping remain central to identifying major-effect loci. For instance, large-scale field trials and genomic analyses pinpointed QT12 on rice chromosome 12; its reduced expression significantly enhances seed-setting rates and yield under heat stress, offering a clear breeding target [100]. As a bridge between genotype and downstream phenotypes, transcriptomic profiling captures the dynamic transcriptional reprogramming of HSFs and HSPs under elevated temperatures [61]. Researchers integrated genome-wide association studies with transcriptomic profiles and identified *TTC4*, a gene encoding a WRKY transcription factor, as a key determinant of heat tolerance in grapevine [82]. Quantitative proteomics enables the identification of differentially abundant proteins under heat stress, particularly those involved in antioxidant defense and protein folding, thereby uncovering the contributions of post-translational modifications and protein–protein interactions to thermotolerance. Metabolites, as the ultimate downstream products of gene expression, serve as direct effector molecules in stress adaptation. Metabolomic profiling has revealed heat-induced accumulation of protective compounds—including proline, betaine, soluble sugars, and phytohormones—which function both as osmotic regulators and as systemic signals. Furthermore, integrative label-free proteomic and metabolomic analyses in heat-stressed grape berries linked heat-accumulated proteins to fruit thermotolerance [101]. The advent of single-cell RNA sequencing has enabled the dissection of heat-response mechanisms at cellular resolution. Application of this single-cell technology to pearl millet leaves showed predominant expression of heat-stress memory genes in vascular cells, demonstrating its ability to resolve cell-type-specific thermotolerance regulators [102].

Translational breeding represents the final frontier of heat-tolerance gene discovery, and CRISPR/Cas9 now serves as the benchmark for both gene validation and resilient germplasm creation. For instance, using prime editing to precisely install heat-responsive elements into cell wall invertase genes increased heat-induced expression in rice and tomato, effectively improving heat-stress yield and climate resilience while preserving fruit quality [103]. However, the application of CRISPR/Cas9 to gene discovery in fruit trees faces major obstacles. Many cultivars exhibit poor responsiveness to tissue culture and low regeneration frequencies, severely hampering the production of edited materials [95]. Currently, agrobacterium-mediated stable transformation remains the predominant method, yet it suffers from low efficiency and strong genotype dependence across most fruit species [104]. Furthermore, the highly heterozygous nature of fruit tree genomes and their extended juvenile periods complicate phenotypic analysis and substantially prolong the time required for trait evaluation [105].

### 5.2. Translational Potential of Current Findings for Orchard Management

Bridging molecular discoveries with practical orchard management, economic returns, and grower decision-making is essential for translating research into climate-adaptive solutions. When molecular mechanisms identify key signaling components involved in heat tolerance, these insights can directly inform management practices. For instance, growers may apply biostimulants or plant growth-promoting rhizobacteria (PGPR) to modulate hormonal balance and antioxidant defenses, thereby delaying leaf senescence and mitigating heat-induced membrane damage and oxidative stress.

Conventional breeding is time-consuming, whereas marker-assisted selection (MAS) and gene editing can markedly accelerate the development of heat-tolerant germplasm. Alternatively, molecularly guided field management can fundamentally alleviate physiological damage under heat stress, preventing substantial yield losses and declines in marketable fruit quality.

Traditional post-harvest yield assessments are inherently retrospective. In contrast, rapid molecular and physiological diagnostic tools enable early, quantitative evaluation of heat damage. Linking these microscale physiological responses to macroscale metrics allows the construction of predictive models linking extreme heat events to physiological injury and subsequent economic losses. Such models would benefit both agricultural insurance agencies in damage assessment and growers in anticipating seasonal revenue fluctuations.

### 5.3. Knowledge Gaps in Heat Tolerance Research of Fruit Trees

Although recent advances have been made in understanding heat tolerance in fruit trees, apple and pear still lag far behind annual model crops like Arabidopsis and rice in terms of knowledge acquisition. First, most studies rely on seedling or root tissues and short-term physiological assays, yet thermotolerance in perennials often diverges between juvenile and mature, fruit-bearing stages; a standardized evaluation system for adult trees is lacking. Second, the storage and retrieval of heat-stress memory during cell division and dormancy remain poorly understood, and the epigenetic mechanisms underlying long-term memory have not been characterized. Third, while roots are known to perceive heat and ABA acts as a long-distance signal, it is unclear how root-zone warming is accurately relayed to the canopy in large trees, and whether peptide signals or small RNAs participate in this systemic communication beyond ABA. Fourth, most studies apply single heat treatments, whereas field heat is often accompanied by high light, drought, or high humidity—conditions that predispose apple and pear to ring rot and anthracnose. How heat suppresses immunity and thereby facilitates disease outbreaks remains an underexplored area warranting further investigation.

### 5.4. Priority Directions for Future Heat-Stress Research on Apple and Pear

Given the current gaps in our understanding of species-specific heat responses, future research on heat tolerance in apple and pear should prioritize the following areas:

1. Heat-responsive regulation of fruit quality. Studies are needed to elucidate how elevated temperatures affect sugar–acid metabolism, anthocyanin biosynthesis, and volatile ester formation, and to determine how heat alters phloem loading and consequently restricts photoassimilate partitioning to developing fruits.
2. Rootstock-mediated signaling and grafting-transmissible thermotolerance. Given that apple and pear are predominantly grafted, rootstocks exert profound effects on scion heat tolerance. Key questions include how thermotolerant rootstocks modulate scion performance via root-derived signals (hormones, mRNAs, proteins) and whether thermotolerance-related signals are translocated across the graft junction. Breeding and selecting heat-tolerant dwarfing rootstocks represents a potentially cost-effective strategy for climate adaptation.
3. Pan-genomic and single-cell resolution germplasm mining. Building single-cell transcriptomic atlases of leaf and root tip tissues will enable precise identification of heat-responsive cell subpopulations and facilitate the discovery of cell-type-specific regulatory factors governing heat tolerance.
4. CRISPR-based breeding integrated with field phenomics. To accelerate translation from laboratory to field, CRISPR/Cas9 editing of promoter regions in negative regulators holds promise for creating heat-resilient germplasm without compromising fruit flavor. Concurrently, deploying UAV-based hyperspectral and thermal imaging can establish high-throughput field phenotyping platforms for large-scale tree populations, thus addressing a major bottleneck in genotype–phenotype association analyses.

## Figures and Tables

**Table 1:** Heat-responsive genes in apple and pear.

Category	Species	Representative Genes	Proposed Function in Heat Response
Transcription factors	Apple/Pear	*MdGATA15*, *MdLBD37*, *PpHsfB2a*	Heat-induced suppressors of anthocyanin biosynthesis via direct repression of key activators or activation of repressors
HSF-HSP chaperone network	Apple	*MdHSFA2*, *MdWRKY75*, *MdEF-Tu*, *MsHsp16.9*, *MdHSP70*	HSFs activate HSPs and chaperones to maintain protein homeostasis and enhance thermotolerance
Nuclear transport	Apple	*MdNup62*, *MdVQ37*	Heat-responsive negative regulators affecting nucleocytoplasmic transport and SA-mediated basal thermotolerance
Cell wall/stress proteins	Apple/Pear	*MdPRP6*, *PpFer1/3/4*	Heat-upregulated structural or protective proteins involved in cell wall integrity and oxidative stress
SUMOylation and epigenetic modifiers	Apple	*MdSUMO2*, *MdSIZ1*, *MdALKBH1A*	Post-translational and epitranscriptomic regulators that fine-tune heat signaling via protein SUMOylation or mRNA m^6^A demethylation
lncRNA-mediated regulatory module	Pear	*HILinc1*/*PbHILT1*/*PbHSFA1b*	lncRNA stabilizes PbHILT1, which enhances PbHSFA1b activity to upregulate downstream heat tolerance genes
Metabolic enzymes	Pear	*SPS*, *SDH*, *SOT*	Heat-driven metabolic reconfiguration affecting sucrose/sorbitol partitioning, watercore, and ripening
Calcium signaling	Pear	*PbCBL2*	Calcium sensor with heat-induced expression, potential role in heat perception
Cell cycle inhibitor	Apple	*MdWEE1*, *MdRBR*, *MdKRPS*	May block the cell-cycle cascade at the G1/S and G2/M transition points under high temperature

## Data Availability

No new data were created or analyzed in this study. Data sharing is not applicable to this article.

## Abbreviations

The following abbreviations are used in this manuscript:

DGK7	Diacylglycerol kinase 7
DAG	Diacylglycerol
PA	Phosphatidic acid
cAMP	Cyclic adenosine monophosphate
ROS	Reactive oxygen species
CNGCs	Cyclic nucleotide-gated channels
CaM	Calmodulin
HSEs	Heat shock elements
HSPs	Heat shock proteins
HDA9	Histone deacetylase 9
HSR	Heat-stress responses
HSFs	Heat shock factors
JA	Jasmonic acid
BRs.	Brassinosteroids
GA	Gibberellin
IAA	Indole-3-acetic acid
ABA	Abscisic acid
MDA	Malondialdehyde
SSC	Soluble solids content
TA	Titratable acidity
H_2_O_2_	Hydrogen peroxide
PPO	Polyphenol oxidase
MBW	MYB-bhlh-WD40
CHS	Chalcone synthase
F3H	Flavanone 3-hydroxylase
FLS	Flavonol synthase
CBL	Calcineurin B-like protein
SPS	Synthase-related genes
SDH	Sorbitol dehydrogenase
SOT	Sorbitol transporter
PRPs	Proline-rich proteins
AGO	Argonaute
m6A	N6-methyladenosine
ASM	Acibenzolar-S-methyl
FKBPs	FK506-binding proteins
AsA	Ascorbic acid
VOCs	volatile organic compounds
SOD	superoxide dismutase
JMJ	Jumonji
CK	cytokinin
MT	Melatonin
GABA	γ-Aminobutyric acid
GAD	Glutamate decarboxylase
GWAS	Genome-wide association studies
PGPR	Plant growth-promoting rhizobacteria
MAS	Marker-assisted selection
